# An Efficient Approach to Screening Epigenome-Wide Data

**DOI:** 10.1155/2016/2615348

**Published:** 2016-03-13

**Authors:** Meredith A. Ray, Xin Tong, Gabrielle A. Lockett, Hongmei Zhang, Wilfried J. J. Karmaus

**Affiliations:** ^1^Division of Epidemiology, Biostatistics, and Environmental Health, School of Public Health, University of Memphis, Zach Curlin Street, Memphis, TN 38152, USA; ^2^Department of Epidemiology and Biostatistics, Arnold School of Public Health, University of South Carolina, 915 Green Street, Columbia, SC 29208, USA; ^3^Human Development and Health, Faculty of Medicine, University of Southampton, 801 South Academic Block Tremona Road, Southampton SO16 6YD, UK

## Abstract

Screening cytosine-phosphate-guanine dinucleotide (CpG) DNA methylation sites in association with some covariate(s) is desired due to high dimensionality. We incorporate surrogate variable analyses (SVAs) into (ordinary or robust) linear regressions and utilize training and testing samples for nested validation to screen CpG sites. SVA is to account for variations in the methylation not explained by the specified covariate(s) and adjust for confounding effects. To make it easier to users, this screening method is built into a user-friendly R package,* ttScreening*, with efficient algorithms implemented. Various simulations were implemented to examine the robustness and sensitivity of the method compared to the classical approaches controlling for multiple testing: the false discovery rates-based (FDR-based) and the Bonferroni-based methods. The proposed approach in general performs better and has the potential to control both types I and II errors. We applied* ttScreening* to 383,998 CpG sites in association with maternal smoking, one of the leading factors for cancer risk.

## 1. Background

Due to its high throughput, accuracy, small sample requirement, and acceptable cost, the Illumina Infinium HumanMethylation450 BeadChip has been widely used to analyze deoxyribonucleic acid (DNA) methylation profiles in epigenetic studies that target various types of cancer. In particular, the illumina infinium assay utilizes a pair of probes (a methylated probe and an unmethylated probe) to measure the intensities of methylated and unmethylated alleles at the interrogated cytosine-phosphate-guanine dinucleotide (CpG) sites [1]. Two measures of DNA methylation are usually used: beta-values and *M*-values. A beta-value is the ratio of signal from the methylated probe relative to the sum of both methylated and unmethylated probes. Beta-values are in the range of (0,1) with 0 being completely unmethylated and 1 being fully methylated. *M*-values are log⁡2 ratio of intensities for methylated and unmethylated probes and range from (−*∞*, +*∞*) [2, 3]. *M*-values are used more often in appreciation of its wide data range and variance homogeneity compared to beta-values.

Given the feature of high dimensionality of high-throughput methylation data, when performing designed and possibly complicated statistical analyses, it is wise to target potentially important CpG sites, for instance, CpG sites potentially associated with single-nucleotide polymorphisms (SNPs) and/or other covariate(s) of interest. Otherwise, the statistical power will be substantially lost. There is evidence that methylation is affected by genetic and some known factors such as smoking [4, 5]. Screening CpG sites have become overwhelmingly important across multiple health fields of study such as cancer research, genetic diseases, and epigenetic research.

Common methods for screening CpG sites assume some relationship of the *M*-values in association with SNPs or some other genetic or environmental factors conditional on the assumption of linearity with some post hoc adjustment for multiple comparisons. The advantage to this method is the flexibility of incorporating additional covariates and their interactions. The primary limitation lies in controlling for multiple testing. Two popular adjustment methods are the Bonferroni-based method [6, 7] and the Benjamini-Hochberg method for controlling the false discovery rate (FDR) [8, 9]. These methods alter the *p*-value or critical value to control for type I error. Bonferroni correction is the most conservative by dividing the linear regression *p*-value, respective of the regression term of interest, by the total number of comparisons (*m*) or CpG sites in this case such that those adjusted *p*-values above the significance level are rejected. The FDR method first orders the *p*-values, *P*(*k*) for *k* ∈ 1 ⋯ *m*, such that lower ordered *p*-values that are less than or equal to *k*/*m* × *α* are rejected [9]. It follows that the conservative Bonferroni-based method cannot control for type II error while the FDR-based method cannot control for type I error.

There are other potential issues that arise when dealing with DNA methylation. It is possible that the variation in methylation cannot be fully explained by the known covariates and there exist latent factors that confound with these known covariates [5]. To improve the screening quality, it is thus important to account for variations introduced by other unknown factors. Furthermore, CpG sites screened from one data set may not be consistent with those from another data set which directly affects the type I error rate and leads to a loss of power. It is thus equally important to improve the reproducibility of the selected CpG sites.

In this paper, we propose a novel collaboration of existing statistical techniques to screen genome-wide methylation. It takes unknown factor effects into account and achieves better reproducibility. The method has the ability to control for both types I and II error while adjusting for covariates as well as latent variables. The proposed screening method incorporates surrogate variable analysis [5], which identifies unknown latent variables, in conjunction with a training and testing approach [10] across CpG methylation sites linearly associated with covariates of interest, including the identified surrogate variables. Independently, each method is well established for different purposes. We mingle these methods and form, compared to existing methods, an improved and more efficient process to screen (filter) informative DNA methylation sites. In addition, this proposed method has been built into an efficient and user-friendly R package. In the following sections, further description and details of the proposed method can be found in Section 2, simulation studies and a real data application are included in Section 3, and we summarize the approach in Section 4.

## 2. Materials and Methods

The proposed screening procedure is built for analyzing the associations between methylation data (*M*-values), or some high-dimensional data, and covariates of interest and their potential interactions. It consists of two consecutive components and surrogate variable analysis followed by a series of regressions while controlling for multiple testing. Surrogate variable analysis (SVA) aims to identify and estimate latent factors or surrogate variables (SVs) that potentially affect the association between known factors and the response variable, for example, SNPs (known factor) and DNA methylation (response variable) [5]. Including the estimated surrogate variables into the screening process has the potential to reduce unexplained variations, adjust for confounding effects, and consequently improve the accuracy of screening in terms of important variable identification [5]. In the context of DNA methylation, inclusion of the surrogate variables explains the variation in DNA methylation not explained by the covariates currently under consideration. This implies that the identified surrogate variables can be further used to identify important factors (markers) showing large contribution to the variation in the response variable explained by the surrogate variables [11]. These surrogate variables along with other variable(s) of interest can be included in regression analysis as independent variables. After SVA, we then begin the screening process with regressions and adjust for multiple comparisons.

As mentioned in the introduction, several methods exist to adjust for multiple testing but lack the ability to control for both types I and II errors. We have elected to implement a method that will control for both while simultaneously helping the issue of reproducibility. We let randomly chosen training and testing samples estimate and test the effects of the primary covariate(s), termed the TT method. General ideas of this approach are discussed in Dobbin and Simon and Faraggi and Simon [10, 12]. This method follows the concept of cross-validation. It has been shown that the implementation of the training and testing technique can provide a better control of type I error rate [10, 12]. In the next two subsections, we provide detailed steps and options available to both the surrogate variable analysis component and the TT method.

### 2.1. Identifying Surrogate Variables

Surrogate variables are inferred prior to screening using an algorithmic method developed by Leek and Storey (2007) called surrogate variable analysis [5]. Following the descriptions in Leek and Storey (2007), these SVs are developed by removing the amount of methylation or signal due to the variable(s) of interest and then decomposing the remaining residuals to identify an orthogonal basis of singular vectors that can be reproduced. These vectors are further examined for significant variation to form surrogate variables. Leek and Storey built an R package to perform the surrogate variable analyses (SVAs). The first step in SVA is to identify the number of surrogate variables based on the data using one of two methods, “be” or “leek” as noted in the package. The “be” method, being the default choice according Leek and Storey's R package, is based on a permutation procedure originally proposed by Buja and Eyuboglu in 1992 [13], while the “leek” method provides an interface to the asymptotic approach proposed by Leek in 2011 [14]. Once the number of surrogate variables is calculated, they are then estimated using one of three algorithms, the iteratively reweighted (“irw”), supervised (“supervised”), and the two-step (“two-step”) method. Iteratively reweighted method is for empirical estimation of control probes, supervised method is for when control probes are known, and the two-step method is for general estimation of surrogate variables [15]. We elected to implement the “two-step” method following Leek and Storey [5]. Conditional on the data, a number of latent unknown variables have been identified, estimated, and will now be incorporated into the regression in association with DNA methylation as additional covariates.

### 2.2. The Training and Testing (TT) Screening Method

After surrogate variable analysis is complete, the TT screening method then begins as an iterative process of randomly sampling the training and testing data by a specified proportion. By default, 2/3 of the data will be included in the training data set, which is suggested in Dobbin and Simon [10] to maximize statistical power. Linear regressions are first applied to the training data to calculate the *p* values for the association between the CpG site and the covariates, including SVs, using either ordinary least squares (this is a default choice in our R package) or robust regression. Robust regression is a type of linear regression that allows for more relaxed assumptions about normality and presence of outliers in the data. A CpG site is included as a candidate if the covariate(s) of interest is statistically significant according to a prespecified significance level, for example, 0.05. In our designed R package, we give user the flexibility to define which term (covariate) is used to decide the selection of CpG sites. For example, suppose the defined right-hand side of the regression is *x*_1_ + *x*_2_ + *x*_1_ × *x*_2_, where *x*_1_ × *x*_2_ denotes the interaction of *x*_1_ and *x*_2_. If the decision of selecting a CpG site is based on one single term, for example, the significance of the interaction effect, the *p* value for the interaction term would be used to test statistical significance against the prespecified significance level of 0.05.

**Figure 1:**
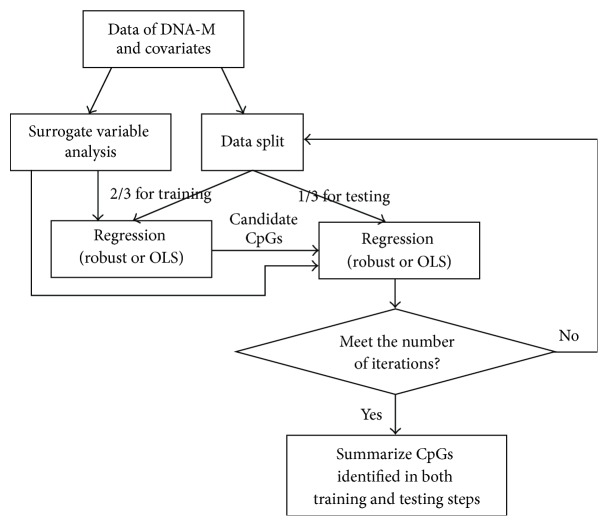
A conceptual representation of the training and testing algorithm.

The process continues with these candidate CpG sites being further tested using the remaining subjects (testing data set) with linear regressions. For one pair of training and testing data sets, a candidate CpG site is deemed as being important if the significance still holds in the testing data. The significance level for the testing data by default is set at the same level as for the training data, 0.05. This screening process will be repeated *i* times (i.e., iterations); at each iteration, a training and testing data set will be randomly selected. After one iteration, a pool of candidate CpG sites will be selected. This process is continued for total *i* iterations. We summarize this screening process in Figure 1. Across all *i* iterations, CpG sites selected in at least *m* iterations will be included in the final pool of potentially important CpG sites; that is, the cutoff percentage of selection is *m*/*i* × 100%. Final estimates of the associations and statistical significance for the selected CpG sites are inferred by use of the complete data via the same analytical methods (i.e., linear regression including surrogate variables previously estimated from complete data) as in the training and testing process. A cutoff percentage of 50 (*m* = 50 across 100 total iterations (*i* = 100)) was used to determine the final pool of potentially important CpG sites. Suggestions on the determination of this predefined value, *m*, are discussed later in Section 3.1.

The user-friendly R package,* ttScreening*, is available from the Comprehensive R Archive Network (CRAN) at https://cran.r-project.org/web/packages/ttScreening/index.html, which implements the proposed screening procedure discussed above. This* ttScreening* package also provides access to other screening methods: FDR and Bonferroni methods. Various options, such as type of linear regression and surrogate variable estimation method, are available for the user to specify while other options are data specific and will need to be defined by the user. However, the package does provide acceptable defaults values for those options that are not data specific. A list of package options along with descriptions are available in the package manual at https://cran.r-project.org/web/packages/ttScreening/ttScreening.pdf.

## 3. Results and Discussion

Simulations are used to demonstrate and assess the TT screening method in comparison with the FDR- and Bonferroni-based methods using the* ttScreening* R package. These are followed up by an application to a data set of 383,998 CpG sites and their association with maternal smoking.

### 3.1. Simulations

*Simulation Scenarios.* In total, 2,000 CpG sites across *n* = 600 subjects were simulated. In these 2,000 CpG sites, *k* sites were assumed to be important. Different settings of *k* were considered, *k* = 10, 100, 200, and 400. Among the *k* important sites, DNA methylation at 90% of the *k* CpG sites was associated with two variables *x*_1_ and *x*_2_, their interaction, and 5 unobservable independent uncorrelated variables, and the remaining 10% of the *k* sites were associated with *x*_1_, the interaction of *x*_1_ and *x*_2_, and the 5 unobservable independent uncorrelated variables. Variable *x*_1_ is generated from normal distribution with mean 1 and variance 1, and *x*_2_ is a four-level categorical variable generated from multinomial with parameters *n* and *π* = {0.15,0.25,0.25,0.35}. The remaining CpG sites (2000 − *k*) were only associated with the 5 unobservable variables and were deemed as unimportant ones. Linear regressions were applied to simulate the data. To assess the robustness of each method (TT screening and FDR- and Bonferroni-based methods), we considered two types of random error in the regressions, one following normal distribution with mean 0 and variance *σ* ^2^ = 1.5 and the other *χ* ^2^ distribution with a degree freedom of 1. Combining the choices on *k* and the distributions of random error, in total, we have 8 settings. For each setting, we generated 100 Monte Carlo (MC) replicates. The results presented below include means of the number of incorrect selections (rounded to the nearest integer), estimates of sensitivity, and estimates of specificity across the 100 MC replicates. The number of incorrect selections refers to the number of CpG sites misidentified (refers to both false positive and false negative CpG sites) out of the 2,000 CpG sites.

### 3.2. Results

Variables *x*_1_ or *x*_2_ would be selected if their interaction effect was statistically significant. All package options were chosen as the default values with training and testing significance levels both set to 0.05. The screening results (Table 1) indicate that, in general, the sensitivity from the FDR-based method is comparable to that from the TT screening method but its specificity is lower when the number of important variables is not sparse; for example, *k* = 200. Compared to the Bonferroni-based method, the TT method in general gave better sensitivity and comparable specificity. These were as expected, as the Bonferroni-based method lacks the ability to control type II error while the FDR-based method cannot control well type I error [16]. The TT screening method, on the other hand, has the potential to control both types I and II errors. This is reflected by the results that the TT screening method overall produced the smallest number of incorrectly identified variables, a statistic incorporating information from both sensitivity and specificity. We also performed the screening using robust regressions and similar results were obtained (not shown). These findings are invariant to the distribution pattern of random errors, normally, or skew distributed.

**Figure 2:**
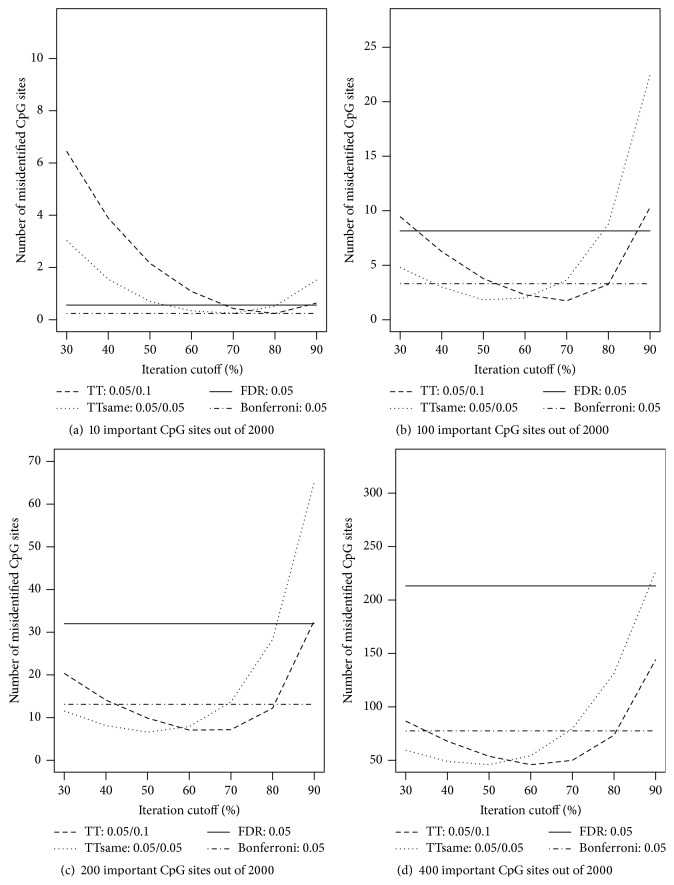
Numbers of misidentified CpG sites versus cutoff frequency for a CpG site being potentially important (based on ordinary least squares regressions). The true numbers of important CpG sites are (a) 10, (b) 100, (c) 200, and (d) 400 out of 2,000 CpG sites. For the TT screening method, two sets of significance levels are considered: (1) 0.05 for training data and 0.1 for testing data; (2) 0.05 for both training and testing data. For the FDR-based and Bonferroni methods, the level was set at 0.05.

Recall that the default value of the cutoff percentage required for a CpG site to be treated as an informative site across all iterations was 50%, and the significance levels for both the training and testing data set were at 0.05. To further evaluate how the choices of this cutoff percentage and the significance levels in the training and testing steps influence the screening results, we chose a set of the cutoff percentage values ranging from 30 to 90 and set the training significance level to 0.1 instead of default value 0.05. All other settings were kept the same. As seen in Figure 2, across the different number of important CpG sites and sample sizes, overall taking the cutoff percentage close to 50% works the best with significance level of 0.05 for both the training and testing steps. If a higher significance level is chosen in the testing step, then a higher value for cutoff percentage should be used.

**Figure 3:**
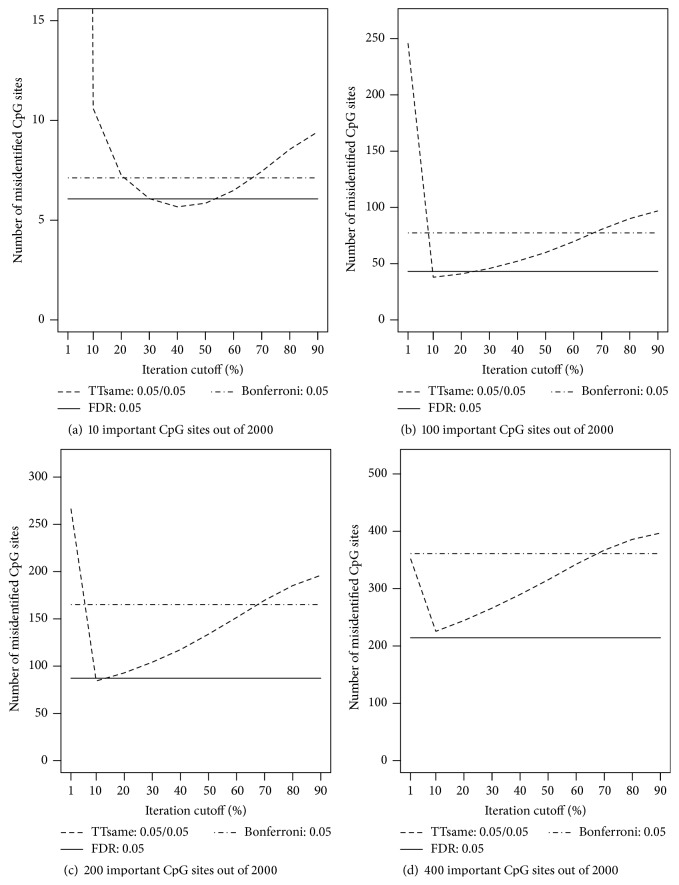
Numbers of misidentified CpG sites versus cutoff frequency for a CpG site being potentially important (based on ordinary least squares regressions). The true numbers of important CpG sites are (a) 10, (b) 100, (c) 200, and (d) 400 out of 2,000 CpG sites across 200 subjects. For the TT screening method, significance levels considered are 0.05 for both training and testing data. For the FDR-based and Bonferroni methods, the level was set at 0.05.

**Figure 4:**
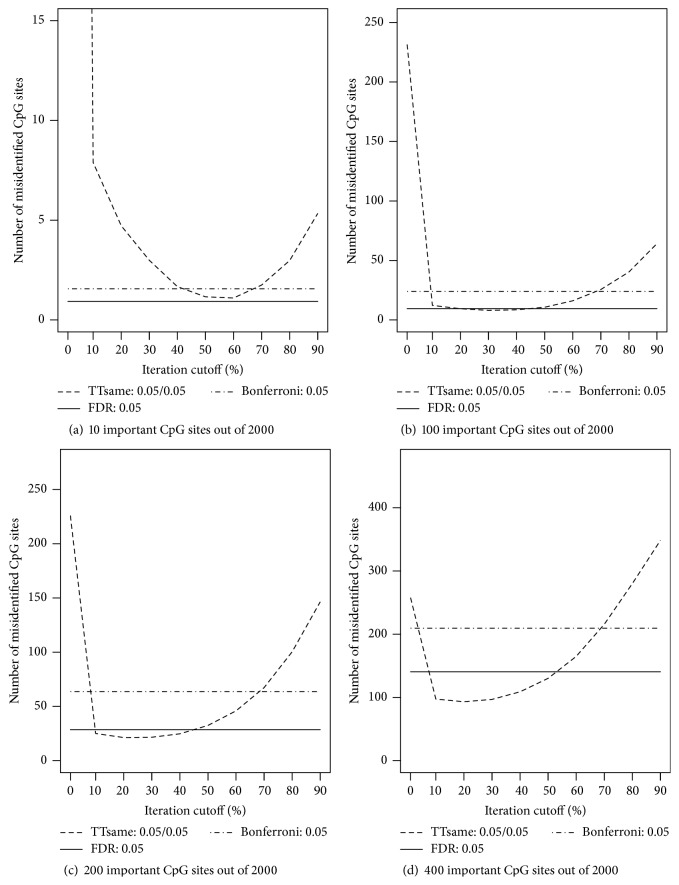
Numbers of misidentified CpG sites versus cutoff frequency for a CpG site being potentially important (based on ordinary least squares regressions). The true numbers of important CpG sites are (a) 10, (b) 100, (c) 200, and (d) 400 out of 2,000 CpG sites across 400 subjects. For the TT screening method, significance levels considered are 0.05 for both training and testing data. For the FDR-based and Bonferroni methods, the level was set at 0.05.

**Figure 5:**
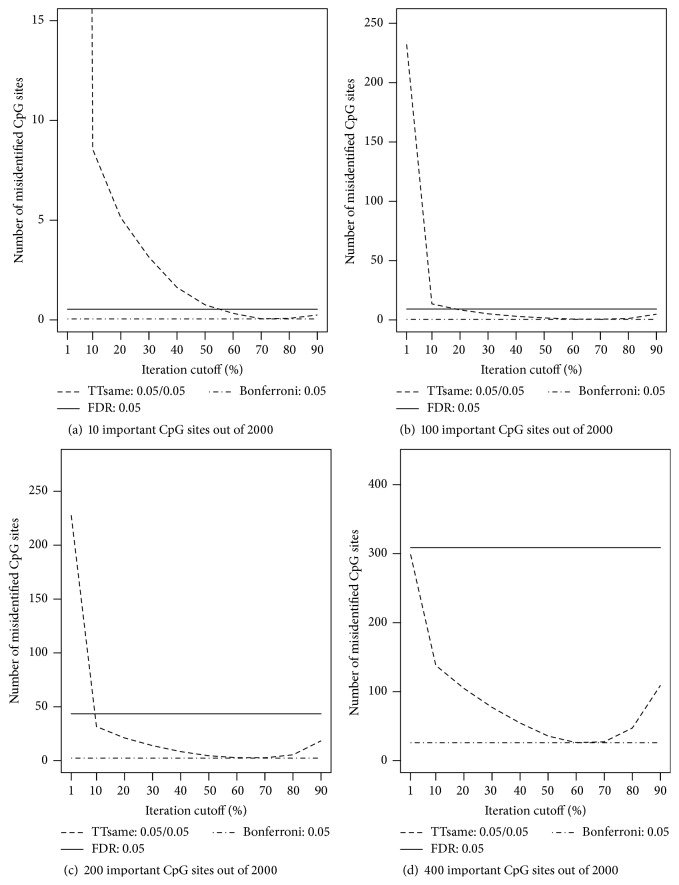
Numbers of misidentified CpG sites versus cutoff frequency for a CpG site being potentially important (based on ordinary least squares regressions). The true numbers of important CpG sites are (a) 10, (b) 100, (c) 200, and (d) 400 out of 2,000 CpG sites across 800 subjects. For the TT screening method, significance levels considered are 0.05 for both training and testing data. For the FDR-based and Bonferroni methods, the level was set at 0.05.

The above examination on cutoff percentage is with sample size *n* = 600. To assess whether and how sample size influences the choice of cutoff percentage, we repeated the above analyses for different sample sizes (and for each sample size, 100 MC replicates were generated), *n* = 200, 400, and 800 with all options in* ttScreening*() set at default values except for cutoff percentage. The results (Figures 3–5) indicated that if the number of important variables is expected to be sparse (e.g., 0.5% of the total number variables), the default cutoff value (50%) is a reasonable choice regardless of the sample size. On the other hand, if important variables are not sparse, for example, at least 5% of the total number of variables, then the cutoff value tends to be influenced by the sample size only if sample size is not large, for example, ≤400. In this case, the smaller the sample size is, the lower the cutoff percentage should be taken. This is as expected; when the sample size is smaller, the probability of true positives will be lower. We thus do not expect a higher proportion of correct selection and consequently a lower cutoff percentage is desired. From our simulations, we recommend 20% cutoff if both the following two conditions are not satisfied: (1) important variables are not expected to be sparse (which rarely happens in high-throughput and high-dimensional data) and (2) we have small samples compared to the number of candidate CpG sites. However, if sample size is large, the default cutoff 50% is still recommended. The selection results for sample sizes *n* = 200, 400, and 800 following these recommendations are included in the appendix (Tables 3, 4, and 5).

To demonstrate the benefit of using the identified surrogate variables in screening, we reanalyzed the simulated data (for *n* = 600) but with the surrogate variable analysis excluded (this option is available in the package). The relative patterns of statistics between different methods were intact compared to the results when surrogate variables were included in the screening process. However, when surrogate variables were not considered, a drastic drop in the mean sensitivity was observed across all the settings (Table 2). The sensitivity measures across all the 100 replicates ranged from 0 to 30%, implying that all methods had trouble deciphering CpG sites that are truly important. However, instead of completely excluding surrogate variables, what if we include a set of surrogate variables with each surrogate variable explaining quite a small portion of variation (i.e., less informative)? To examine this, we extracted surrogate variables that are less informative and reanalyzed the data. Similar findings as shown in Table 2 were concluded (Table 6), indicating the importance of including informative surrogate variables in order to substantially improve the quality of selection.

We further examined confounding effect of surrogate variables. To achieve this, we followed the same simulation scenarios noted earlier but, instead of assuming independence between all the (observed and unobserved) independent variables, we allow the 5 unobserved variables to be correlated with observed variable *x*_1_ with a correlation of 0.7^|*i* − *j*|^ where *i*, *j* ∈ *L* and *L* = (0,1, 2,3, 4,5) represents an arbitrary locations of the variables. For example, the observed variable is identified at location 0 and the 5 unobserved variables are locations (1,2, 3,4, 5). Then we generated 100 Monte Carlo replicates based on normal distributions with a sample size of *n* = 600. In the simulations, we considered the impact of including and excluding identified surrogate variables. The screening results (results not shown) are consistent with the previous findings when no correlations between unobserved and observed variables were assumed (Tables 1 and 2); that is, including the identified surrogate variable substantially improves the screening statistics (number of incorrectness, sensitivity, and specificity).

Based on the simulations, it is advised that users follow default setting for the TT screening method: 2/3 of the data for training, “two-step” for SVA analysis as described in the published literature [5], 100 iterations for the total number of TT screenings, 50% as the cutoff proportion of those 100 iterations, and 0.05 significance level for the training and testing data. We recommend 100 iterations as a balance between computing efficiency and adequate resampling of the subjects to decipher true associations. We recommend 50% as the default because in general informative CpGs are sparse compared to the number of candidate CpG sites, in which case, as seen in simulations the 50% cutoff percentage is suitable for small and large sample sizes.

### 3.3. Real Data Analysis

We applied the* ttScreening* package to 383,998 CpG sites with DNA methylation available for 245 subjects. The data were collected from a study cohort of 18-year-old females on the Isle of Wight (IOW) in the United Kingdom [17]. We examined a single factor that may be potentially associated with DNA methylation, maternal smoking status during pregnancy (0/1). It is thought that maternal smoking in utero increases chances of asthma and wheezing in children and modifies defensive mechanisms such as xenobiotic detoxification systems, antioxidant responses, and damage repair mechanisms [18]. Certain modifications to such systems have been known to increase risk of lung cancer [18].

We applied the TT, FDR-based, and Bonferroni-based methods to identify potentially important CpG sites. In the TT method, 2/3 of the samples were used for training and the remaining for testing. The number of iterations was set at 100 with relative frequency of 50% as the cutoff and the significance level in both training and testing steps was set at 0.05. The significance level for FDR-based and Bonferroni-based methods was set to 0.05. OLS regression was used to estimate associations and *p* values. Other settings were chosen as the default.

**Figure 6:**
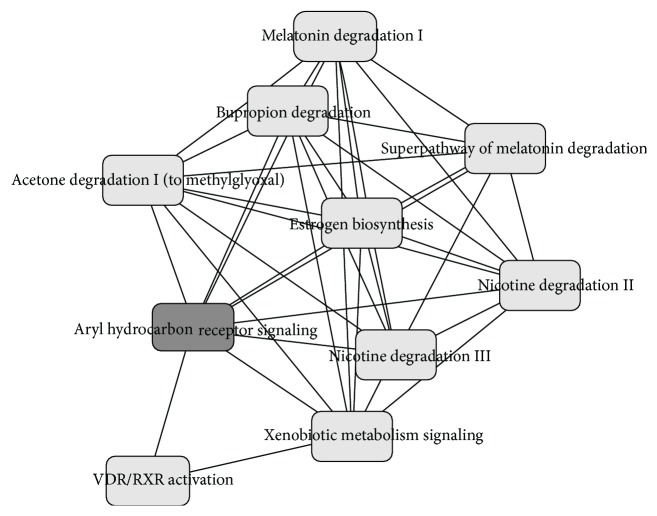
Top pathways enriched among genes identified by training and testing method from the Isle of Wight data include aryl hydrocarbon receptor signaling and xenobiotic metabolism signaling, which overlap with other pathways including the nicotine degradation II and nicotine degradation III pathways, as well as pathways for the metabolism of cigarette smoke components and producing known effects of smoke exposure.

FDR- and Bonferroni-based methods, respectively, identified ten and five CpG sites associated with maternal smoking status. The five CpG sites identified by the Bonferroni-based method were also included in the ten CpG sites identified by FDR. The TT screening method identified 91 CpG sites potentially linked to maternal smoking status. The 10 CpG sites collectively identified by Bonferroni and FDR were included in the 91 identified by TT. Significant CpG site locations and annotated genes identified are included in Table 7 in Figure 6.

To understand the biological meaning of the identified sets of CpGs, pathway analysis was used. The genes annotated to each significant CpG site were extracted from the 450 K array manifest file (v1.2; available: http://support.illumina.com/downloads/humanmethylation450_15017482_v1-2_product_files.html). Where a CpG site was annotated to more than one gene, all annotated genes were included. The resulting gene lists were analyzed with Ingenuity Pathway Analysis tool (IPA; Qiagen). Statistical significance for each pathway is reported by IPA using *p* values. The set of 91 CpGs differentially methylated with maternal smoking identified by TT (includes the 5 and 10 CpG sites identified by Bonferroni and FDR, resp.) were mapped to 54 unique genes.

The 91 CpGs differentially methylated with maternal smoking included 18 previously identified top maternal smoking-associated CpG sites in* AHRR*,* CYP1A1*,* GFI1*,* MYO1G*,* CNTNAP2* [4, 19–21],* FRMD4A* [4, 21],* LRRC32*, and intergenic CpGs near* LOC284998* and* PDE10A-SDIM1* [21]. Bonferroni identified five maternal smoking-associated CpGs and FDR identified ten (including all five identified by Bonferroni), all of which have been previously published in association with maternal smoking. However, the TT method identified many more CpGs that have been previously identified as statistically significant in other cohorts, suggesting that these are not simply false positive results, and represent additional truly maternal smoking associated genes worthy of future investigation and validation. Our observation of differential methylation at age 18 in response to maternal smoking during pregnancy also agrees with previous observations that these epigenetic responses are preserved at least into adolescence [20]. The top pathways enriched among genes containing the 91 CpG sites included aryl hydrocarbon receptor signaling (*p* = 0.005) and xenobiotic metabolism signaling (*p* = 0.030), which support a long-lasting effect of maternal smoking on metabolic pathways controlling responses to smoke exposure. Furthermore, these pathways overlap with other pathways including the nicotine degradation II and nicotine degradation III pathways, as well as pathways for the metabolism of cigarette smoke components, and producing known effects of smoke exposure (Figure 6), such as melatonin degradation [22]. The TT method also identified six differentially methylated CpG sites in* HOXA2*, whose mutation causes cleft palate [23], the risk of which is known to be affected by maternal smoking [21].

In conclusion, the CpGs detected by TT in association with maternal smoking were enriched among pathways related to the known biology of those processes. Disruption or modification of some of these pathways results in greater risk of lung cancer [18]. TT also identified CpGs located in genes not previously identified which indicates potentially new findings.

## 4. Conclusions

We developed a unique screening procedure built into an R package for the purpose of screening important variables. It includes the developed method that involves training and testing steps (the TT screening method), along with another two existing methods, the method controlling for FDR and the other controlling overall significance level through the Bonferroni method. Simulations are used to demonstrate and assess the TT screening method.

Overall, the TT screening method produced comparable sensitivity results to that of FDR-based method and comparable specificity results of Bonferroni-based methods. However, the number of misidentified CpG sites from the TT method is in general smaller than those from the other two approaches. The findings on sensitivity and specificity were as expected, because the Bonferroni-based method lacks the ability to control type II error while the FDR-based method cannot control well type I error [16]. The TT screening method, on the other hand, has the potential to control both the types I and II errors, which was supported by the smaller numbers of incorrect detections. It was also noticed that, by incorporating surrogate variable analysis, all three methods produced higher sensitivity measures. In the real data application, the TT method identified a larger number of CpGs compared to the other two methods. The subsequent pathway analyses further support the practical strength of the proposed method. It is worth noting that since the screening process is to identify the CpG sites, to properly assess the functionality of these sites, it is critical to evaluate them jointly, for example, using pathway analyses.

An important contribution of the developed process is its computing efficiency. Although we combined three methods into one package, the computing is not burdensome. The computing time for screening 383,998 CpG sites (the real data analysis) with the* ttScreening*() function using default options only takes 82 minutes on a personal computer with a central processing unit (CPU) of 2.0 gigahertz (GHz) and memory of 4.0 gigabytes (GB) of random-access memory (RAM). The* ttScreening*() function always automatically adjusts for multiple testing using three methods, FDR, Bonferroni, and the training and testing method.

Lastly, the versatility of the proposed screening method allows it to be applied to a variety of scientific fields in which large data or high dimensionality is a computational problem. DNA methylation is of growing interest in all aspects of public health, in cancer and genetics/epigenetics specifically. TT reduces dimensionality in a timely manner while controlling for types I and II errors and adjusting unknown latent variables estimated using the surrogate variable analysis. Finally, the package incorporates a variety of options which allows the user to create very specific settings while maintaining convenient usability.

## Figures and Tables

**Table 1:** Simulation results for selecting *k* important variables among 2,000 candidates with surrogate variables included.

Statistics	Bon	FDR	TT	Bon	FDR	TT
Random error normally distributed						

	*k* = 10			*k* = 100		
# incorrect	0	1	1	3	8	2
Sensitivity	0.981	0.991	0.994	0.968	0.999	0.991
Specificity	1	1	1	1	0.996	1

	*k* = 200			*k* = 400		
# incorrect	13	32	7	78	213	46
Sensitivity	0.936	0.998	0.982	0.814	0.996	0.931
Specificity	1	0.982	0.998	0.998	0.868	0.989

Random error *χ* ^2^ distributed (df = 1)						

	*k* = 10			*k* = 100		
# incorrect	1	1	1	16	9	7
Sensitivity	0.892	0.958	0.963	0.837	0.982	0.941
Specificity	1	1	1	1	0.996	1

	*k* = 200			*k* = 400		
# incorrect	46	29	21	169	158	95
Sensitivity	0.771	0.981	0.906	0.582	0.969	0.790
Specificity	1	0.986	0.999	0.999	0.909	0.993

Bon: Bonferroni, FDR: false discovery rate, and TT: training and testing.

**Table 2:** Simulation results for selecting *k* important variables among 2,000 candidates with surrogate variables excluded.

Statistics	Bon	FDR	TT	Bon	FDR	TT
Random error normally distributed						

	*k* = 10			*k* = 100		
# incorrect	10	51	9	99	114	90
Sensitivity	0.011	0.054	0.100	0.014	0.167	0.102
Specificity	1	0.979	1	1	0.984	1

	*k* = 200			*k* = 400		
# incorrect	197	182	180	394	310	359
Sensitivity	0.015	0.232	0.102	0.015	0.288	0.104
Specificity	1	0.984	1	1	0.984	1

Random error *χ* ^2^ distributed (df = 1)						

	*k* = 10			*k* = 100		
# incorrect	10	38	9	98	103	91
Sensitivity	0.018	0.053	0.095	0.015	0.158	0.096
Specificity	1	0.986	1	1	0.99	1

	*k* = 200			*k* = 400		
# incorrect	197	176	181	394	314	363
Sensitivity	0.014	0.217	0.094	0.014	0.273	0.095
Specificity	1	0.989	1	1	0.986	1

Bon: Bonferroni, FDR: false discovery rate, and TT: training and testing.

**Table 3:** Simulation results for selecting *k* important variables among 2,000 candidates including surrogate variables across 200 subjects.

Statistics	Bon	FDR	TT	Bon	FDR	TT
Random error normally distributed						

	*k* = 10			*k* = 100^†^		
# incorrect	7	6	6	77	43	41
Sensitivity	0.292	0.423	0.475	0.227	0.605	0.641
Specificity	1	1	1	1	0.998	0.997

	*k* = 200^†^			*k* = 400^†^		
# incorrect	165	87	93	361	214	244
Sensitivity	0.175	0.616	0.571	0.099	0.565	0.432
Specificity	1	0.994	0.996	1	0.975	0.989

Random error *χ* ^2^ distributed (df = 1)						

	*k* = 10			*k* = 100^†^		
# incorrect	9	8	7	88	62	55
Sensitivity	0.126	0.193	0.307	0.124	0.408	0.498
Specificity	1	1	1	1	0.998	0.997

	*k* = 200^†^			*k* = 400^†^		
# incorrect	182	123	119	380	274	288
Sensitivity	0.093	0.422	0.435	0.051	0.377	0.314
Specificity	1	0.996	0.997	1	0.985	0.992

Bon: Bonferroni, FDR: false discovery rate, and TT: training and testing. ^†^Cutoff percentage of 20%.

**Table 4:** Simulation results for selecting *k* important variables among 2,000 candidates including surrogate variables across 400 subjects.

Statistics	Bon	FDR	TT	Bon	FDR	TT
Random error normally distributed						

	*k* = 10			*k* = 100^†^		
# incorrect	2	1	1	24	10	9
Sensitivity	0.851	0.949	0.941	0.761	0.968	0.965
Specificity	1	1	1	1	0.997	0.997

	*k* = 200^†^			*k* = 400^†^		
# incorrect	64	29	21	210	141	93
Sensitivity	0.682	0.968	0.947	0.479	0.948	0.864
Specificity	1	0.988	0.994	0.999	0.925	0.976

Random error *χ* ^2^ distributed (df = 1)						

	*k* = 10			*k* = 100^†^		
# incorrect	4	3	3	47	19	18
Sensitivity	0.612	0.767	0.786	0.531	0.878	0.883
Specificity	1	1	1	1	0.997	0.997

	*k* = 200^†^			*k* = 400^†^		
# incorrect	111	42	42	289	146	141
Sensitivity	0.445	0.88	0.837	0.281	0.841	0.717
Specificity	1	0.99	0.995	0.999	0.949	0.983

Bon: Bonferroni, FDR: false discovery rate, and TT: training and testing. ^†^Cutoff percentage of 20%.

**Table 5:** Simulation results for selecting *k* important variables among 2,000 candidates including surrogate variables across 800 subjects.

Statistics	Bon	FDR	TT	Bon	FDR	TT
Random error normally distributed						

	*k* = 10			*k* = 100		
# incorrect	2	44	5	26	309	36
Sensitivity	0.991	1	0.998	0.949	1	0.988
Specificity	1	0.976	0.998	0.996	0.807	0.981

	*k* = 200			*k* = 400		
# incorrect	64	29	32	210	141	130
Sensitivity	0.682	0.968	0.847	0.479	0.948	0.696
Specificity	1	0.988	0.999	0.999	0.925	0.995

Random error *χ* ^2^ distributed (df = 1)						

	*k* = 10			*k* = 100		
# incorrect	0	1	1	4	9	2
Sensitivity	0.975	0.994	0.993	0.961	0.998	0.99
Specificity	1	1	1	1	0.995	0.999

	*k* = 200			*k* = 400		
# incorrect	15	34	7	82	214	50
Sensitivity	0.928	0.998	0.98	0.803	0.994	0.924
Specificity	1	0.981	0.998	0.998	0.868	0.988

Bon: Bonferroni, FDR: false discovery rate, and TT: training and testing.

**Table 6:** Simulation results for selecting *k* important variables among 2,000 candidates including the most and least important surrogate variables across 600 subjects.

	Bonferroni			FDR			TT		
	*n*.sv = 5	*n*.sv = 10	*n*.sv = 15	*n*.sv = 5	*n*.sv = 10	*n*.sv = 15	*n*.sv = 5	*n*.sv = 10	*n*.sv = 15
Most important surrogate variables included									

	# incorrect								
*k* = 10	0	0	0	6	6	6	5	4	3
*k* = 100	2	3	2	19	19	19	7	5	5
*k* = 200	6	6	5	26	29	29	6	6	4
*k* = 400	12	11	11	40	38	39	7	7	7

	Sensitivity								
*k* = 10	1	1	1	1	1	1	1	1	1
*k* = 100	0.98	0.97	0.98	1	1	1	0.98	0.98	0.98
*k* = 200	0.97	0.97	0.975	0.995	0.995	0.995	0.985	0.985	0.985
*k* = 400	0.97	0.973	0.973	1	1	1	0.988	0.988	0.985

	Specificity								
*k* = 10	1	1	1	0.997	0.997	0.997	0.997	0.998	0.998
*k* = 100	1	1	1	0.99	0.99	0.99	0.997	0.998	0.998
*k* = 200	1	1	1	0.986	0.984	0.984	0.998	0.998	0.999
*k* = 400	1	1	1	0.975	0.976	0.976	0.999	0.999	0.999

Most important surrogate variables not included									

	# incorrect								
*k* = 10	10	10	10	10	10	10	10	10	10
*k* = 100	100	100	100	100	100	100	100	100	100
*k* = 200	200	200	200	200	200	200	200	200	200
*k* = 400	400	400	400	400	400	400	400	400	400

	Sensitivity								
*k* = 10	0	0	0	0	0	0	0	0	0
*k* = 100	0	0	0	0	0	0	0	0	0
*k* = 200	0	0	0	0	0	0	0	0	0
*k* = 400	0	0	0	0	0	0	0	0	0

	Specificity								
*k* = 10	1	1	1	1	1	1	1	1	1
*k* = 100	1	1	1	1	1	1	1	1	1
*k* = 200	1	1	1	1	1	1	1	1	1
*k* = 400	1	1	1	1	1	1	1	1	1

FDR: false discovery rate, TT: training and testing, *n*.sv = number of surrogate variables, and *k*: the number of truly important CpG sites out of 2,000.

**Table 7:** Information for CpG sites associated with maternal smoking identified by the training and testing screening method.

Model 2: maternal smoking only				
CpG site	Chromosome: map	Gene	Promoter region	CpG island
name	information	name	and exon	location
cg20464068	chr1: 14026054	*PRDM2*	TSS1500	14026481–14027200
cg07951355	chr1: 40123717			
cg14179389	chr1: 92947961	*GFI1*	Body	92945907–92952609
cg00909806	chr1: 212688762			212688031–212688448
cg26143053	chr2: 3718125	*ALLC*	5′UTR	
cg03690080	chr2: 39188006	*LOC100271715, LOC375196*	Body, TSS1500	39186777–39187968
cg19243656	chr2: 73340094	*RAB11FIP5*	5′UTR, 1stExon	73339292–73340733
cg18703066^‡^	chr2: 105363536			
cg07516970	chr2: 157181359	*NR4A2*	3′UTR	157184389–157184632
cg19273101	chr2: 191734576			
cg14075934	chr2: 200137014	*SATB2*	Body	
cg03158780	chr3: 577964			
cg10663973	chr4: 6642559	*MRFAP1*	1stExon, 5′UTR	6642194–6643322
cg10364374	chr4: 125857940			
cg23743778	chr4: 140357491			140357362–140357610
cg21401642	chr4: 174421114			174421347–174421559
cg17924476^†^	chr5: 323794	*AHRR*	Body	320788–323010
cg05575921	chr5: 373378	*AHRR*	Body	373842–374426
cg21161138	chr5: 399360	*AHRR*	Body	
cg12287936	chr5: 1800606	*NDUFS6, MRPL36*	TSS1500	1799461–1801905
cg16244648	chr5: 141555043			
cg18349863	chr6: 29912713	*HLA-A*	Body	29910202–29911367
cg11492288	chr6: 30290596	*HCG18*	Body	30294169–30295071
cg04325960	chr6: 147124986	*LOC729176, C6orf103*	TSS200, Body	
cg00004963	chr6: 147124996	*LOC729176, C6orf103*	TSS200, Body	
cg11881038	chr6: 154408701	*OPRM1*	Body, 1stExon, 5′UTR	
cg20418529	chr6: 166260012			
cg00794911	chr6: 166260532			
cg18132363^†^	chr6: 166260572			
cg08634229	chr6: 169326603			
cg06769202	chr7: 27142535	*HOXA2*	TSS200	27143181–27143479
cg23206851	chr7: 27143046	*HOXA2*	TSS1500	27143181–27143479
cg02225599	chr7: 27143252	*HOXA2*	TSS1500	27143181–27143479
cg10319053	chr7: 27143370	*HOXA2*	TSS1500	27143181–27143479
cg00445443	chr7: 27143478	*HOXA2*	TSS1500	27143181–27143479
cg06401979	chr7: 27143717	*HOXA2*	TSS1500	27143181–27143479
cg11986226	chr7: 40026390	*CDK13*	Body	
cg19089201^‡^	chr7: 45002287	*MYO1G*	3′UTR	45002111–45002845
cg04180046^‡^	chr7: 45002736	*MYO1G*	Body	45002111–45002845
cg12803068^‡^	chr7: 45002919	*MYO1G*	Body	45002111–45002845
cg25949550^‡^	chr7: 145814306	*CNTNAP2*	Body	145813030–145814084
cg11207515	chr7: 146904205	*CNTNAP2*	Body	
cg21015808	chr7: 149809179			
cg21330896	chr7: 28205705	*ZNF395*	3′UTR	
cg17199018	chr7: 28206278	*ZNF395*	Body	
cg04690729	chr8: 133494328	*KCNQ3*	TSS1500	133492398–133493586
cg15707110	chr8: 144311102			144311708–144311985
cg08126560	chr9: 92291523	*LOC100129066*	Body	92291268–92291524
cg13393408	chr9: 132874232	*GPR107*	Body	
cg11813497	chr10: 14372879	*FRMD4A*	TSS200	
cg12490835	chr10: 22623821			22623350–22625875
cg26520012	chr10: 42672589			42672509–42673432
cg05329352	chr10: 112838983	*ADRA2A*	1stExon	112835990–112839303
cg18424850	chr10: 132945786	*TCERG1L*	Body	
cg19494188	chr11: 1466780	*BRSK2*	Body	1466304–1467210
cg26204383	chr11: 2435667	*TRPM5*	Body	2435295–2436651
cg14436038	chr11: 6494706	*TRIM3*	5′UTR	6494725–6495453
cg15627089	chr11: 16625751			16626053–16629180
cg25160605	chr11: 21087846	*NELL1*	Body	
cg17517598	chr11: 61659090	*FADS3*	TSS200	61658569–61659592
cg10788371	chr11: 76381040	*LRRC32*	5′UTR, 1stExon	76381449–76382295
cg11395306	chr11: 98939366	*CNTN5*	5′UTR	
cg01186919	chr11: 111742365	*ALG9*	TSS1500, TSS200	111741953–111742292
cg02820646	chr11: 115398838			
cg05730269	chr11: 118477055	*PHLDB1*	TSS200, TSS1500	118478235–118481896
cg18493761	chr11: 125386885			
cg09932758	chr12: 58022542	*B4GALNT1*	Body	58021294–58022037
cg09644707	chr12: 114885161			114885105–114885418
cg27103591	chr12: 124809023	*NCOR2*	3′UTR	124808972–124809176
cg02032696	chr14: 67982198	*TMEM229B*	TSS200	67981514–67982380
cg21511816	chr14: 76597824			76597648–76597911
cg24874277	chr15: 33211107	*FMN1*	Body	
cg03643241	chr15: 44487910	*FRMD5*	TSS1500	44486741–44487860
cg20596162	chr15: 45408861	*DUOXA2*	Body	45408573–45409528
cg16754378	chr15: 57179394	*LOC145783*	Body	57179277–57179838
cg06899985	chr15: 65689298	*IGDCC4*	Body	65689142–65689362
cg05549655^†^	chr15: 75019143	*CYP1A1*	TSS1500	75018186–75019336
cg17852385	chr15: 75019188	*CYP1A1*	TSS1500	75018186–75019336
cg11924019^†^	chr15: 75019283	*CYP1A1*	TSS1500	75018186–75019336
cg18092474^†^	chr15: 75019302	*CYP1A1*	TSS1500	75018186–75019336
cg01060282	chr16: 17033575			
cg07675285	chr16: 27121267			27121011–27121241
cg11705699	chr16: 87742845	*KLHDC4*	Body	87742556–87743109
cg09554007	chr16: 89627174	*RPL13, SNORD68*	5′UTR, 1stExon, TSS1500	89626644–89627869
cg16483033	chr17: 1090441	*ABR*	1stExon, 5′UTR	
cg08682866	chr17: 13818946			
cg17624073	chr17: 79393583	*BAHCC1*	Body	79393341–79393742
cg13723693	chr17: 80656731	*RAB40B*	TSS200	80655335–80657183
cg18495341	chr17: 80909836	*B3GNTL1*	Body	
cg18449879	chr19: 16045054	*CYP4F11*	1stExon, Body	
cg02543506	chr20: 33876594	*FAM83C*	Body	33879904–33880215

CpG: cytosine-phosphate-guanine dinucleotide, chr: chromosome, locations of each CpG are for v37 of the human genome, ^†^CpG sites identified by false discovery rate, and ^‡^CpG sites identified by false discovery rate and Bonferroni-based methods.

## Appendix

See Tables 3, 4, 5, 6, and 7 and Figures 3, 4, 5, and 6.
